# Editorial: Deciphering molecular mechanisms behind tumor heterogeneity features by using nucleic acid-based technologies

**DOI:** 10.3389/fgene.2026.1917362

**Published:** 2026-07-14

**Authors:** Alexis Germán Murillo Carrasco, José Luis Buleje Sono, Manuel Gentiluomo, Mateus Nóbrega Aoki, Ricardo Fujita, Tatiane Katsue Furuya

**Affiliations:** 1 Centro de Investigação Translacional em Oncologia (LIM24), Instituto do Câncer do Estado de São Paulo (ICESP), Faculdade de Medicina da Universidade de São Paulo (FMUSP), São Paulo, Brazil; 2 Comprehensive Center for Precision Oncology, Universidade de São Paulo (USP), São Paulo, Brazil; 3 Centro de Genética y Biología Molecular (CGBM), Instituto de Investigación, Facultad de Medicina Humana, Universidad de San Martín de Porres, Lima, Peru; 4 Department of Biology, University of Pisa, Pisa, Italy; 5 Laboratory of Molecular Oncology, Carlos Chagas Institute, Oswaldo Cruz Foundation (Fiocruz), Curitiba, Brazil

**Keywords:** liquid biopsy, multiomics, precision oncology, profiling technologies, tumor heterogeneity

Over the last decade, our understanding of the molecular mechanisms underlying cancer has evolved substantially. We now recognize that tumors are not homogeneous entities, even within a single patient. Rather, they comprise multiple cellular and molecular subclones that coexist, compete, and evolve, potentially harboring intrinsic resistance mechanisms or acquiring resistance during treatment (Marusyk et al., 2020). This dynamic evolutionary landscape transforms cancer therapy into a continuous “whack-a-mole” challenge, in which the elimination of one resistant population often enables the emergence of others. Therefore, deciphering tumor heterogeneity has become one of the main goals of cancer research and precision oncology.

A critical aspect of this effort involves uncovering the molecular regulators that drive cellular plasticity and adaptation. Among these regulators, non-coding RNAs (ncRNAs) have emerged as key players in the control of gene expression and cellular fate. In this Research Topic, the review of Moraes Reis and Sanchez Bassères discusses the involvement of long non-coding RNAs (lncRNAs) in regulatory processes, including alternative splicing, microRNA (miRNA) processing, and peptide-associated functions. These mechanisms challenge the classical interpretation of the central dogma of molecular biology, providing new insights into how regulatory networks contribute to cancer stemness, phenotypic plasticity, and tumor evolution.

The implementation of emerging genomic technologies has enabled the growing recognition of such molecular complexity in tumor biology. High-throughput sequencing and advanced molecular profiling approaches have transformed our ability to characterize tumors at unprecedented resolution. Wallen et al. demonstrated the utility of RNA hybrid-capture-based sequencing for a more accurate assessment of *NTRK* gene-associated fusions, enabling not only the identification of clinically relevant alterations but also the discovery of novel fusion variants with potential relevance for targeted therapies.

Another transformative technology reshaping cancer research has been single-cell sequencing, which has dramatically increased the resolution at which the tumor microenvironment can be studied. By resolving tumors at the level of individual cells, these approaches have provided a deeper understanding of cellular dynamics, lineage relationships, and functional heterogeneity within tumors. Nevertheless, their implementation in routine research and clinical settings remains challenging, particularly in underrepresented populations (https://pubmed.ncbi.nlm.nih.gov/41106359/), where access to frozen-tissue biobanks and standardized workflows is limited. Addressing this gap, Yan et al. presented promising results from evaluating a novel single-cell probe-based sequencing strategy using locally archived Formalin-fixed Paraffin-embedded (FFPE) samples. Their findings demonstrated the feasibility of extracting single-cell information from clinically accessible specimens, expanding new opportunities for retrospective studies and broader population representation in cancer research.

The clinical relevance of these advances becomes particularly evident when investigating the molecular mechanisms underlying tumors that arise at younger ages. Early-onset malignancies have attracted growing attention because of their increasing incidence and frequently aggressive clinical behavior. In this Research Topic, Nguyen et al. analyzed the transcriptomic landscape of colorectal cancer and identified molecular features associated with early-onset disease. Their findings suggested that these tumors may exhibit a more aggressive phenotype and poorer prognosis, even among patients diagnosed at early clinical stages. Functional analyses further linked these observations to alterations in mTOR signaling, cell-cycle regulation, and apoptosis pathways, emphasizing the value of molecular profiling for understanding disease behavior and identifying new therapeutic vulnerabilities.

As our capacity to characterize tumor heterogeneity improves, there is an increasing need for minimally invasive strategies capable of capturing these molecular dynamics over time. In this context, liquid biopsy has emerged as a promising tool for cancer detection, monitoring, and treatment guidance. Schwab and Nonaka reviewed the potential application of miRNAs as diagnostic, prognostic, and predictive biomarkers in gastrointestinal malignancies, highlighting their relevance across a range of biofluids for early detection and precision medicine.

The relevance of liquid biopsy extends beyond solid tumors and is becoming increasingly evident in hematopoietic malignancies, where continuous disease monitoring is particularly important. Yang et al. described a case of anaplastic lymphoma kinase-positive anaplastic large cell lymphoma (ALK + ALCL), in which whole-exome sequencing enabled the identification of the *NPM1::ALK* gene fusion, illustrating the clinical value of comprehensive molecular characterization. Subsequently, Shi et al. investigated diffuse large B-cell lymphoma (DLBCL) and identified mutations potentially associated with early treatment failure following frontline R-CHOP chemo-immunotherapy, providing insights that may support risk stratification and therapeutic decision-making. Expanding the concept of circulating biomarkers even further, Wosniaki et al. characterized the proteomic cargo of extracellular vesicles (EVs) in leukemia patients according to treatment resistance status and the presence of clinically relevant mutations, including *ABL1* variants associated with resistance to tyrosine kinase inhibitors (TKIs).

Taken together, the studies presented in this Research Topic illustrate how advances in molecular biology, sequencing technologies, and liquid biopsy approaches are improving our understanding of tumor heterogeneity. These studies demonstrate how emerging technologies can bridge the gap between biological discovery and clinical implementation. By uncovering novel molecular mechanisms and biomarkers, while enabling more comprehensive characterization of tumor evolution through both tissue- and blood-based analyses, these contributions provide valuable insights for patient stratification, disease monitoring, and the continued development of precision oncology strategies.
